# Supplementary material for: Gut microbiome and plasma metabolome alterations in ileostomy and after closure of ileostomy
Source: Microbiol Spectr. 2025 Mar 4;13(4):e01191-24. doi: 10.1128/spectrum.01191-24 (PMC11960061; doi:10.1128/spectrum.01191-24)
Supplement: Supplemental figure legends — Legends for Figures S1 to S6. [file spectrum.01191-24-s0001.docx]

**Figure 1** Grouping of participants and flow chart. The flow chart details the disposal methods, sample types, and collection time points of phases E0, E1 and E2. Groups are marked with corresponding colours.

**Figure 2** Comparison of species diversity before and after ileostomy closure. **A.** Dilution curve. The horizontal coordinate represents the number of tags extracted, and the ordinate indicates that the diversity index value was calculated when the corresponding number of tags was extracted. **B.** α diversity index. From left to right are Shannon, Simpson, Chao1 and Sob. **C.** β diversity analysis model. The left and right sides are the PCoA and NMDS analyses. **D.** ANOSIM of gut microbes. R=0.5477 and p=0.001, indicating a moderate statistical difference among the three groups. The level of species analysis above is at the OTU, and the distance is bray. Repeated measures ANOVA combined with Tukey test were used in B plot, * signifies p-value＜0.05, ** indicates p-value＜0.01, *** signifies p-value＜0.001, **** means p value ＜0.0001, ns represents no significant difference. **Abbreviation:** *PCoA* principal coordinate analysis, *NMDS* non-metric multidimensional scaling analysis, *ANOSIM* analysis of similarity, *OTU* operational taxonomic unit.

**Figure 3** Analysis of gut microbial composition before and after ileostomy closure at group level. **A.** Relative abundance of gut microbes stack maps. The left diagram shows the difference in composition at the phylum level, while the right diagram shows the genus level. The horizontal axis represents the grouping, and the vertical axis represents the relative abundance of species. **B.** Circos maps of flora distribution. The left and right diagrams are the phylum and genus level circos maps, respectively. One side of the graph is the group information, and the other side is the species information. The lines on both sides indicate a pair of corresponding relationships, and the thicker the lines, the larger the abundance value.

**Figure 4** Indicator flora genera and functional prediction at different phases. **A.** Venn analysis plot of gut microbes. When the average tag value is > 1, the bacteria genus in the group is considered to exist. The diagram presents the number of common and unique bacteria among the groups. **B.** Enriched ternary graph of gut microbes. The different dot colours represent the different enriched groups. Different dots represent different genera, and the size of the dots indicates the average abundance of the genus. The location of the points is constituted by the relative abundance share of the species in the three groups. The top 10 bacteria genera with the highest average abundance are annotated. **C.** Bubble plot of IndVal analysis. This plot reveals the enrichment of the top 10 genera by IndVal in their respective periods. The colour of the dots represents the different groups, and the size of the dots represents the IndVal. **D.** Phenotypic abundance heat map. 9 types of gut microbes' phenotypic abundance are presented. **E.** Heat map of functional abundance. Dynamically displaying the function distribution of different groups. **F.** The correlation network of gut microbes between phases E1 and E2. The dot colour represents the different enriched groups, and the dot size indicates the number of correlations. The dashed line represents the negative correlation, the solid line indicates the positive correlation, and the line thickness represents the strength of the correlation.

**Abbreviation:** *IndVal* indicator value.

**Figure 5** Global targeted metabolites and signalling pathway association analysis. **A.** Clustering heat map of DAMs. The horizontal coordinate represents a sample from each individual, and the vertical coordinate represents the name of each DAM. The colour of the block represents the standardized relative abundance of the metabolite. The upper colour bar represents the sample grouping. The left colour bar represents the metabolite classification. **B.** VIP plot of DAMs. VIP > 1.000, p-value < 0.05. **C.** Correlation heatmap of DAMs. Correlation analysis was conducted by calculating Pearson’s correlation coefficient for two metabolites. Positive and negative correlations are indicated by the red and blue dots, respectively. **D.** Network diagram of metabolites and associated signalling pathways. Blue dots represent metabolite enrichment pathways, and other dots represent metabolites. The size of the blue dot indicates the number of molecules associated with it, and those of the other dots indicate the size of the log2(FC) value by gradients. **E.** Bubble diagram of the enriched pathways of DAMs. The horizontal axis and the size of the dots represent the impact value, and the vertical axis and the colour of the dots represent the p-value of the hypergeometric distribution test of KEGG enrichment. Repeated measures ANOVA adopted in Figure A screened DAMs. **Abbreviation:** *DAM* differentially abundant metabolites, *VIP* variable importance for the projection, *KEGG* Kyoto encyclopaedia of genes and genomes, *FC* fold change, *ANOVA* analysis of variance.

**Figure 6** Analysis of the association between gut microbial genera and plasma metabolites. **A.** O2PLS loading diagram of gut microbes and plasma metabolites. Plasma metabolites are on the left, and gut microbes are on the right. The horizontal and vertical axes are the first and second dimensional coordinates, respectively. The greater the absolute value of the coordinates of these dots, the greater the degree of correlation. The top 10 are represented by red dots. **B.** Integrated O2PLS loading diagram. The top 25 loading values (the sum of squares of loading values 1 and 2) of plasma metabolites and gut microbes were screened. **C.** Correlated heat map of gut microbes and plasma metabolites. The horizontal and vertical axes are the gut microbes and DAMs, respectively. The DAMs in red font indicate that they overlap in Figure5A. The colour gradient of the square is the size of the correlation coefficient. On the left and at the top are cluster trees for the row and column data, respectively, with asterisks in the cells indicating correlation significance p-values. * means p-value＜0.05, ** means p-value＜0.01, *** means p-value ＜0.001. **D.** Correlation network diagram of the gut microbes and plasma metabolites. The relation pairs with absolute correlation coefficient values of > 0.5 are screened. The red squares and blue dots represent the gut microbes and plasma metabolites, respectively. The solid and dashed lines represent positive and negative correlations, respectively. **Abbreviation:** *DAM* differentially abundant metabolites, *O2PLS* bidirectional orthogonal projections to latent structures model.
