# Supplementary material for: Gut microbiome and plasma metabolome alterations in ileostomy and after closure of ileostomy
Source: Microbiol Spectr. 2025 Mar 4;13(4):e01191-24. doi: 10.1128/spectrum.01191-24 (PMC11960061; doi:10.1128/spectrum.01191-24)
Supplement: Supplemental figures and tables — Tables S1 to S3; Figures S1 to S8. [file spectrum.01191-24-s0002.pdf]

Name	Function content	Version
VennDiagram	Draw Venn diagram	1.6.16
psych	Calculation and testing of inter-species correlation coefficients	1.8.4
circlize	Draw Circos diagrams	0.4.12
igraph	Map of interspecies correlation networks	1.1.2
ggtren	Ternary plot	3.1.0
labdsv	Indicator analysis	2.0-1
Vegan	Bray distance index/ PCA/ PCoA/ NMDS/ Anosim/ Welch 's t test	2.5.3
ggplot2	Draw stack diagram/ PCA/ PCoA/ NMDS diagram/ dilution curve	2.2.1
ropls	Multivariate statistical analysis/ PCA/ PLS-DA/ OPLS-DA	1.20.0
pheatmap	Heat mapping	1.0.12
OmicsPLS	Multi-omics association analysis/ O2PLS model	2.0.2

Table S1 Information about used R language packages. **Abbreviation:** *PCA* principal component analysis, *PCoA* principal coordinate analysis, *NMDS* non-metric multidimensional scaling analysis, *PLS-DA* partial least squares-discriminant analysis, *OPLS-DA* orthogonal partial least squares discriminant analysis, *O2PLS* bidirectional orthogonal projections to latent structures model.

Feature name		
**Gender**		
Male	66.7%	20 admissions
Female	33.3%	10 admissions
**Body figure**		
Height	162(150-175)	centimeter
Weight	60(47-83)	kilogram
BMI	22.8(18.4-31.6)	
**Age**	58(33-81)	years
**Ileostomy duration**	179(53-581)	days
**Operative reason**		
Malignant tumor	93.3%	28 admissions
Benign tumor	6.7%	2 admissions
**Comorbidity**		
Hypertension	20.9%	6 admissions
Diabetes	13.3%	4 admissions
**Complication**		
Incision infection	6.7%	2 admissions

Table S2 Basic information of patients. **Abbreviation:** *BMI* body mass index.

DMA name	Class	Changes	VIP	p value	FDR
hydroquinone	Xenobiotics	Decrease	1.03	3.22E-12	9.87E-12
thymine	Nucleotide	Decrease	1.87	3.16E-12	9.74E-12
xanthine	Nucleotide	Decrease	1.53	7.45E-10	1.32E-09
hypoxanthine	Nucleotide	Decrease	1.36	8.56E-13	3.27E-12
(r)-5,6-dihydrothymine	Nucleotide	Decrease	1.27	1.17E-08	1.79E-08
malonate	Lipid	Increase	1.30	1.85E-12	4.77E-12
sphingosine	Lipid	Increase	1.14	5.97E-09	9.43E-09
traumatic Acid	Lipid	Decrease	1.62	2.60E-09	4.29E-09
ethylmethylacetic acid	Lipid	Decrease	1.62	5.04E-12	1.15E-11
cholesterol	Lipid	Decrease	1.35	7.31E-11	1.54E-10
riboflavin	Cofactors and Vitamins	Decrease	1.03	1.45E-07	2.02E-07
2-ketobutyric acid	Carbohydrate	Increase	1.52	1.35E-11	3.43E-11
maleic acid	Carbohydrate	Increase	1.42	5.27E-17	2.88E-15
sucrose	Carbohydrate	Decrease	1.96	4.17E-12	1.24E-11
succinic acid	Carbohydrate	Decrease	1.93	6.85E-18	3.14E-15
l-fucose	Carbohydrate	Decrease	1.08	4.30E-11	9.52E-11
l-rhamnono-1,4-lactone	Carbohydrate	Decrease	1.06	4.24E-10	7.83E-10
l-leucine	Amino acid	Increase	1.89	9.60E-17	7.62E-15
creatine	Amino acid	Increase	1.70	3.69E-13	1.69E-12
se-Methylselenocysteine	Amino acid	Increase	1.52	2.61E-09	4.30E-09
alpha-ketoisovaleric acid	Amino acid	Decrease	1.67	9.62E-07	1.19E-06
l-glutamine	Amino acid	Decrease	1.48	4.89E-09	7.04E-09
4-hydroxycinnamoylagmatine	Amino acid	Decrease	1.41	9.11E-16	1.75E-14
pyroglutamic acid	Amino acid	Decrease	1.30	7.52E-12	2.08E-11
pyrrolidonecarboxylic acid	Amino acid	Decrease	1.17	1.41E-06	1.82E-06
pyrrolidonecarboxylic acid	Amino acid	Decrease	1.17	1.41E-06	1.82E-06

Table S3 Statistical table of DAMs. **Abbreviation:** *DAM* differentially abundant metabolites, *VIP* variable importance for the projection, *FDR* false discovery rate.

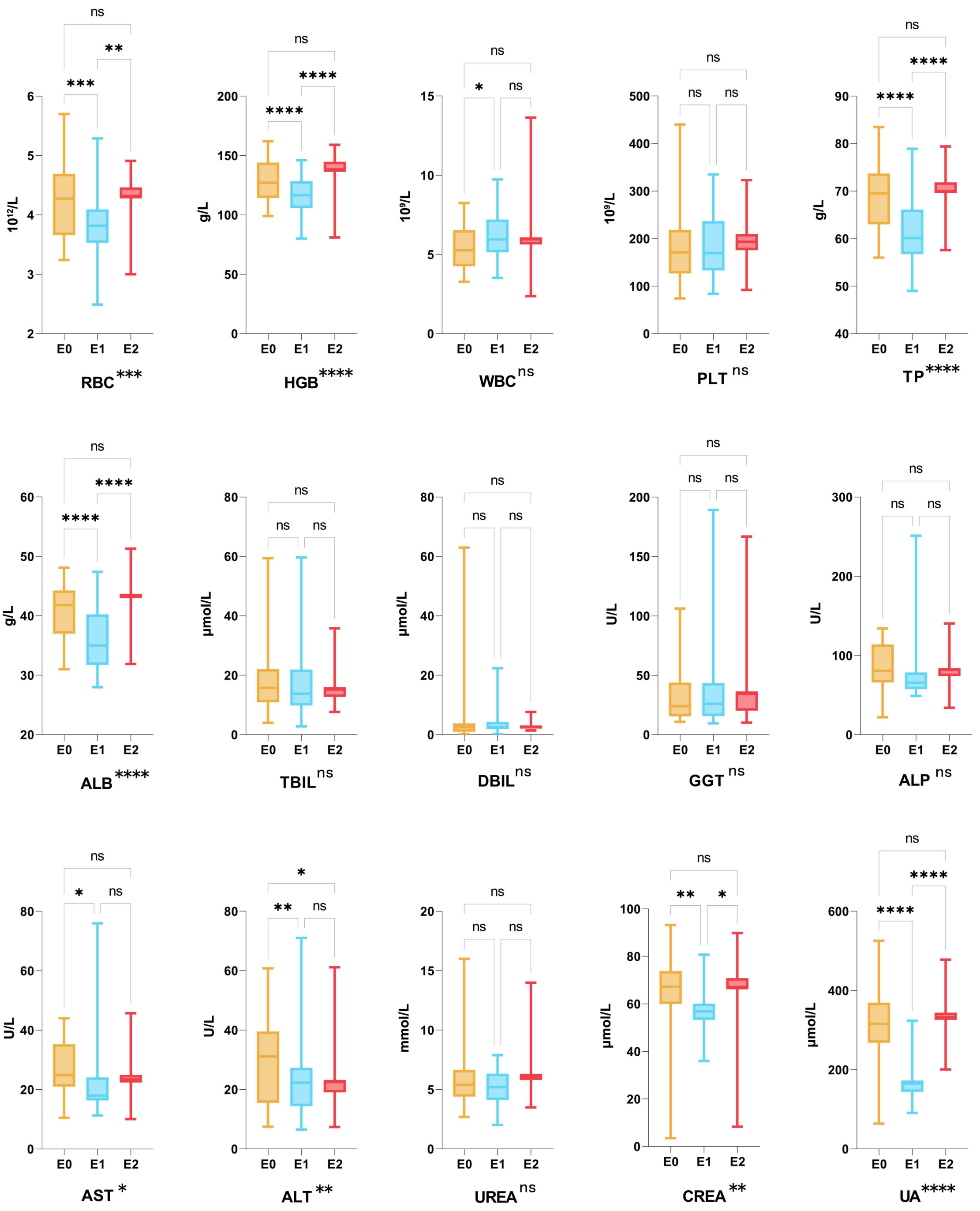

Figure S1 Changes of clinical indicators before and after closure of ileostomy. Repeated measures ANOVA combined with Tukey test were used, * means p value$<$0.05, ** means p value $<$0.01, *** means p value $<$0.001, **** means p value $<$0.0001, ns means no significant difference. **Abbreviation:** *ANOVA* analysis of variance, *RBC* red blood cell count, *HGB* hemoglobin, *WBC* white blood cell count, *PLT* platelet, *GGT* γ-glutamyltransferase, *DBIL* direct bilirubin, *CREA* plasma creatinine, *TP* plasma total protein, *TBIL* total bilirubin, *AST* aspartate aminotransferase, *ALT* alanine aminotransferase, *ALB* plasma albumin, *ALP* alkaline phosphatase, *UREA* blood urea, *UA* plasma uric acid.

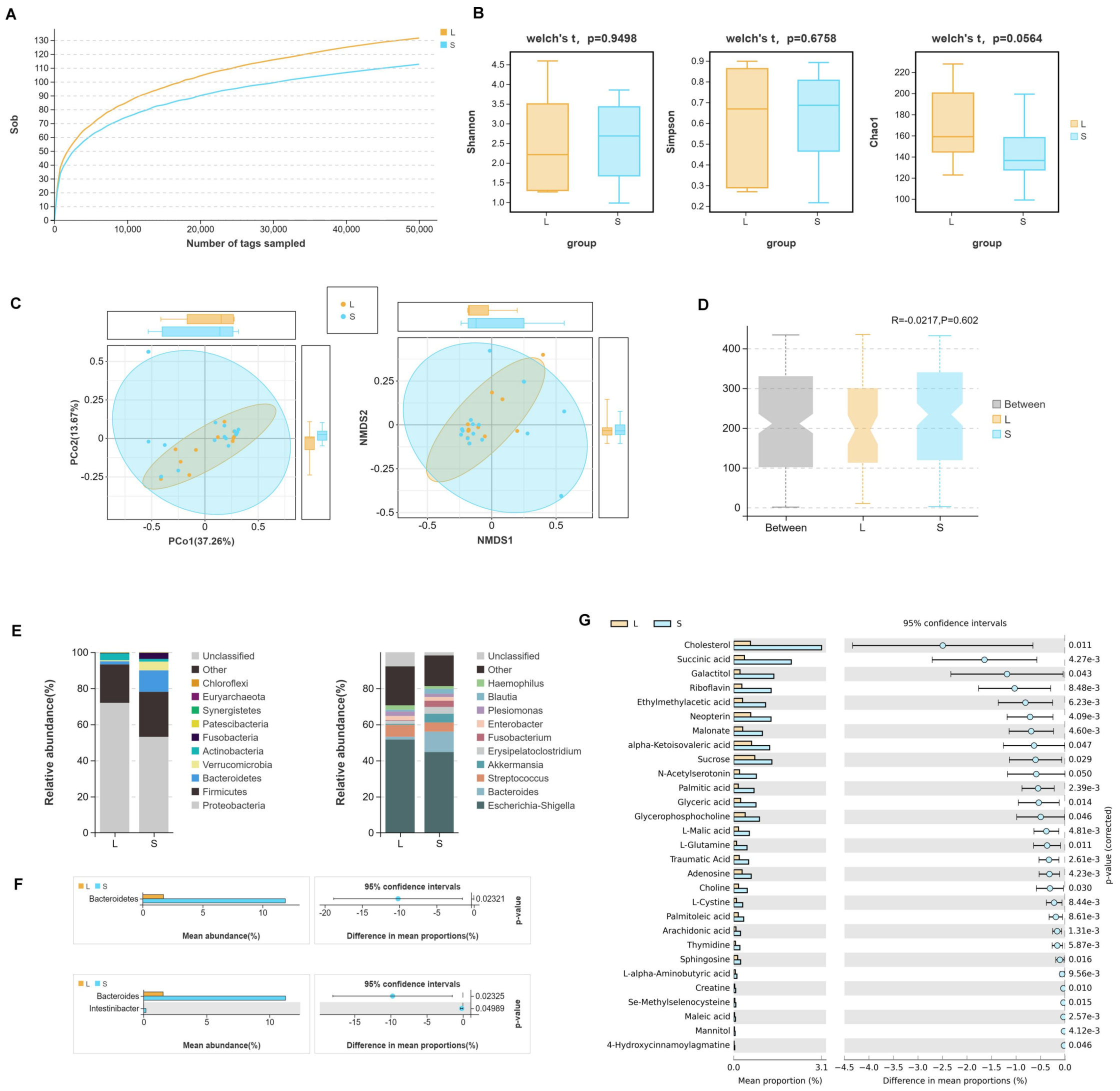

Figure S2 Effects of different duration of ileostomy on microflora and metabolites. **A.** Dilution curve. The horizontal coordinate represents the number of tags extracted, and the ordinate indicates that the diversity index value was calculated when the corresponding number of tags was extracted. The curve flattens out and the sequencing volume can be considered sufficient. **B.** α diversity index. From left to right are Shannon, Simpson, and Chao1, with no significant difference between the two groups. **C.** β diversity analysis model. The left and right sides are the PCoA and NMDS analyses respectively. **D.** ANOSIM of gut microbes. R=-0.0217 and p=0.602, indicating no statistical difference between the two groups. **E.** Relative abundance of gut microbes stack map. The legend shows the names of the major gut microbes, with phylum level on the left and genus level on the right. **F.** Pairwise comparison of gut microbes. The legend shows the gut microbe names with statistical differences, above and below are at the phylum and genus level respectively. **G.** Pairwise comparison of DAMs. Welch's t test was used in diagram B, F and G. The structure of the F and G diagram is the same. In the left half of each plot, the vertical coordinate represents the differential names, and the horizontal coordinate represents the average abundance. The horizontal coordinate of the right half is the difference in abundance between groups, the color of the points represents the group with higher abundance, the error bar of the points represents the fluctuation range of the 95% confidence interval of the difference, and the vertical coordinate represents the significance of the difference between the corresponding groups,

that is, the size of the p value. In the above grouping, L represents the long-term group of 180 days with ileostomy or more, and S, conversely, represents the short-term group. **Abbreviation:** *PCoA* principal coordinate analysis, *NMDS* non-metric multidimensional scaling analysis, *ANOSIM* analysis of similarity, *OTU* operational taxonomic unit.

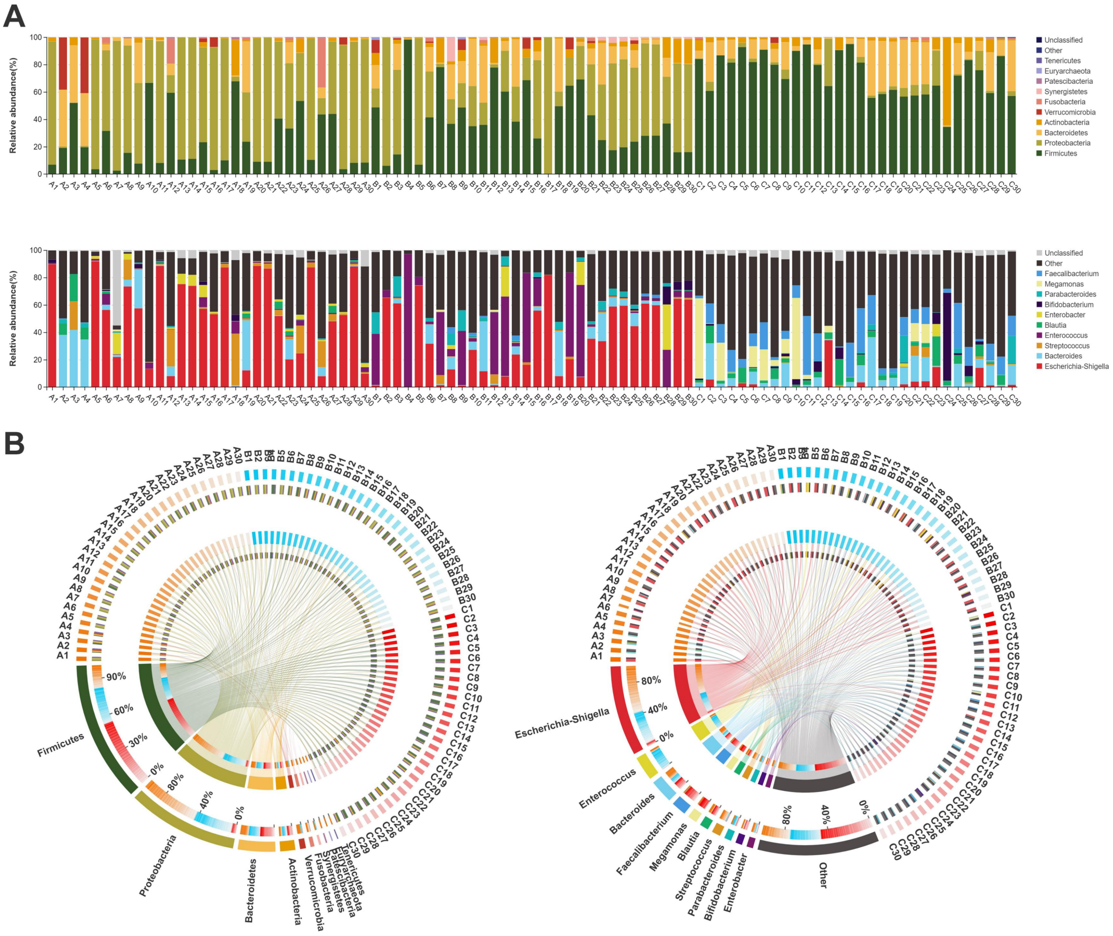

Figure S3 Analysis of gut microbial composition before and after ileostomy closure at sample level. **A.** Relative abundance of gut microbes stack map. The diagrams illustrate the relative abundance of each sample, while An, Bn, and Cn at the bottom represent patients from phases E0, E1, and E2, respectively. The above and below diagrams show the classification of phylum and genus levels respectively. **B.** Circos map of flora distribution. The left and right diagrams are the phylum and genus level circos maps, respectively.

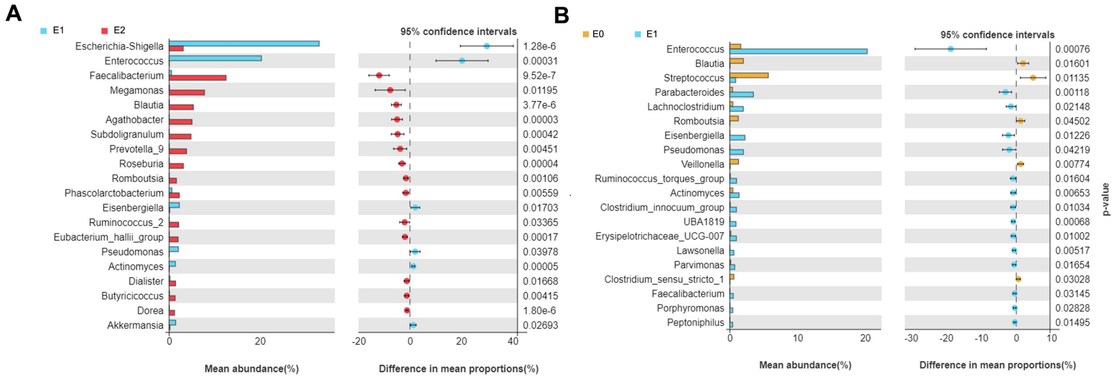

Figure S4 Pairwise comparison of bacterial genera. **A.** Comparison between phases E1 and E2. **B.** Comparison between phases E0 and E1. Welch's t test was used in this diagram. In the left half of each plot, the vertical coordinate represents the differential gut microbial genera, and the horizontal coordinate represents the average abundance. The horizontal coordinate of the right half is the difference in abundance between groups, the color of the points represents the group with higher abundance, the error bar of the points represents the fluctuation range of the 95% confidence interval of the difference, and the vertical coordinate represents the significance of the difference between the corresponding groups, that is, the size of the p value.

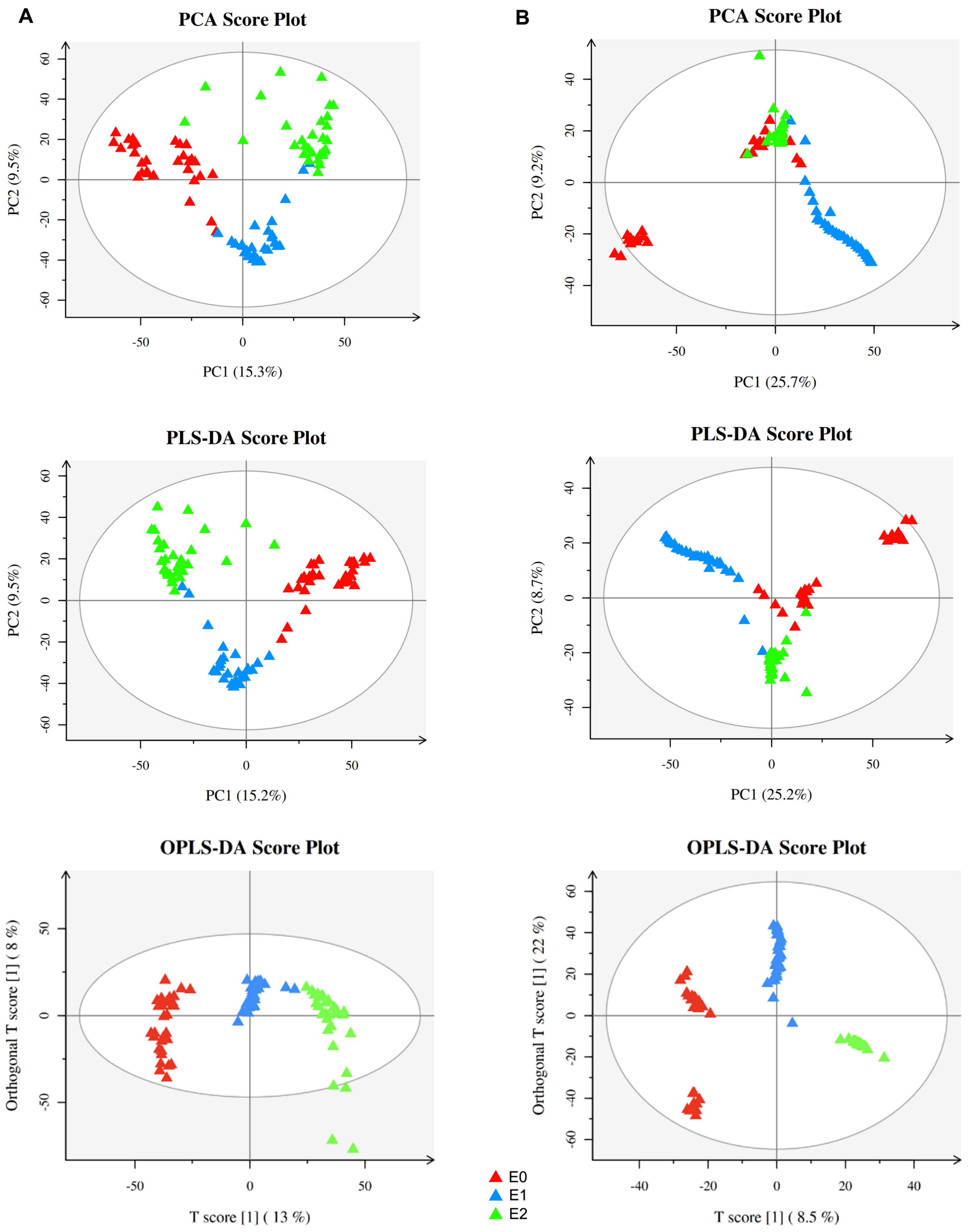

Figure S5 Score map of multivariate statistical analysis. **A.** Cationic mode. **B.** Anion mode. **Abbreviation:** *PCA* principal component analysis, *PLS-DA* partial least squares-discriminate analysis, *OPLS-DA* orthogonal projections to latent structures discriminant analysis.

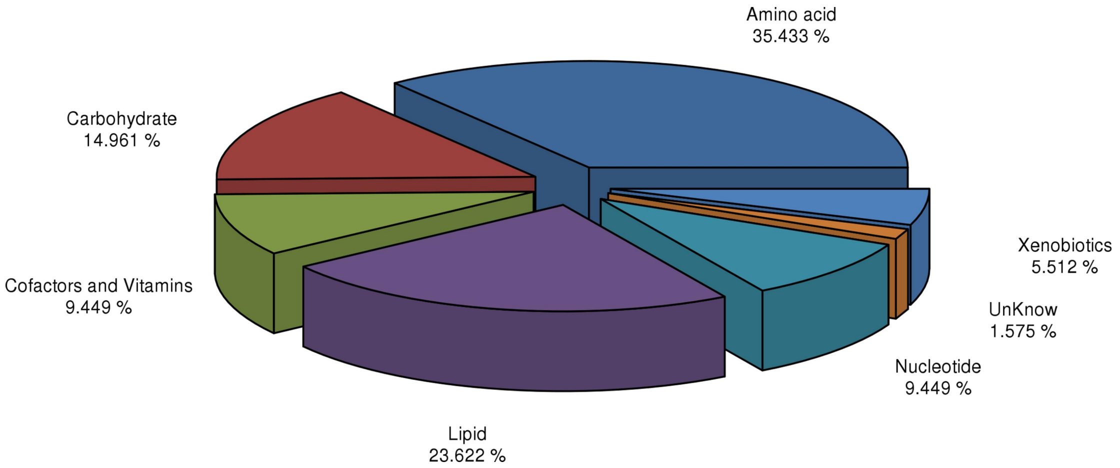

Figure S6 Pie chart for complete identification of metabolites.

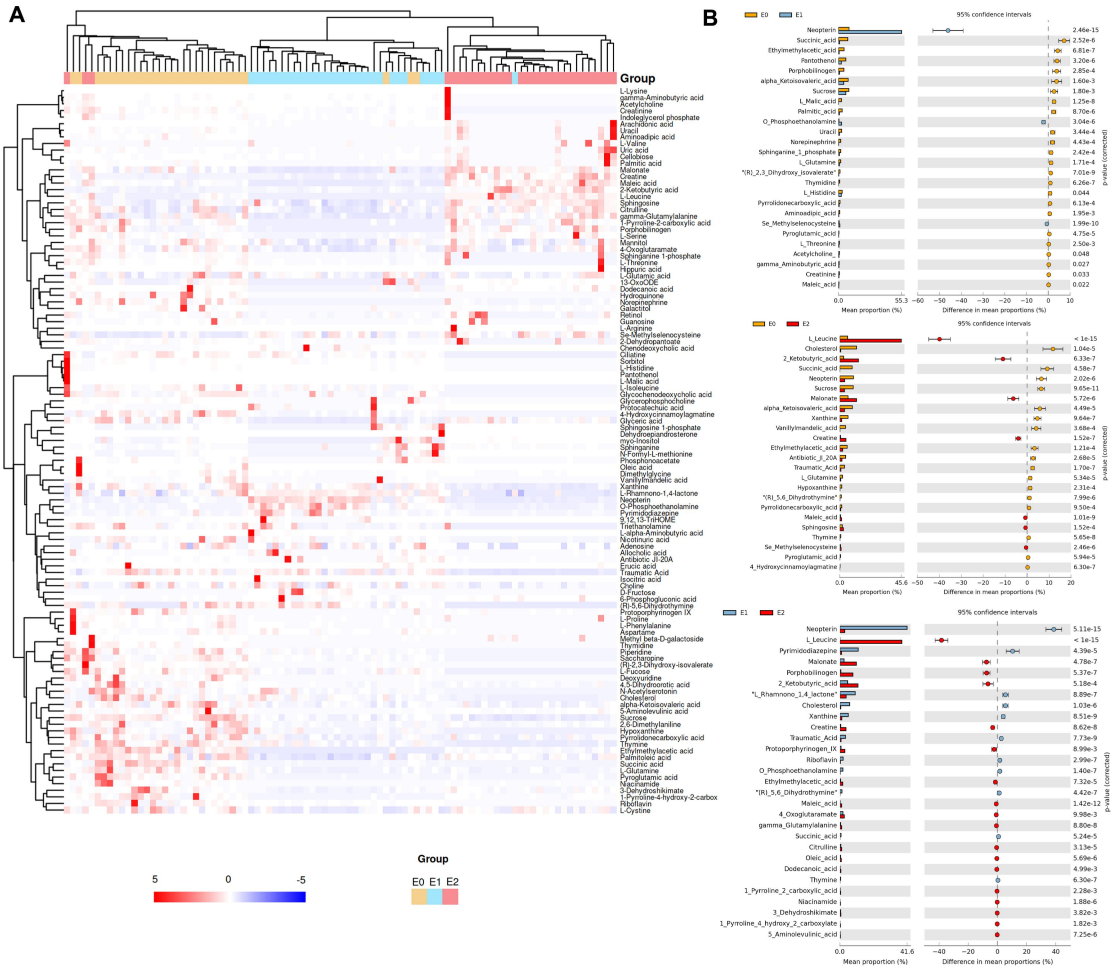

Figure S7 All DAMs obtained by pairwise comparison methods. **A.** Clustering heat map of all DAMs. The horizontal coordinate represents each sample individual, and the vertical coordinate represents the name of each DAM. This figure uses agglomerate hierarchical clustering to classify each object step by step to obtain similar clustering. The color of the block represents the standardized relative abundance of the metabolite. The upper colored bar represents the actual grouping of samples. **B.** Pairwise comparison of DAMs by Welch's t test. From top to bottom, E0 and E1, E0 and E2, and E1 and E2 are the DAMs obtained by pairwise comparison. In the left half of each plot, the vertical coordinate represents the differential DAMs, and the horizontal coordinate represents the average abundance. The horizontal coordinate of the right half is the difference in abundance between groups, the color of the points represents the group with higher abundance, the error bar of the points represents the fluctuation range of the 95% confidence interval of the difference, and the vertical coordinate represents the significance of the difference between the corresponding groups, that is, the size of the p value. **Abbreviation:** *DAM* differentially abundant metabolites.

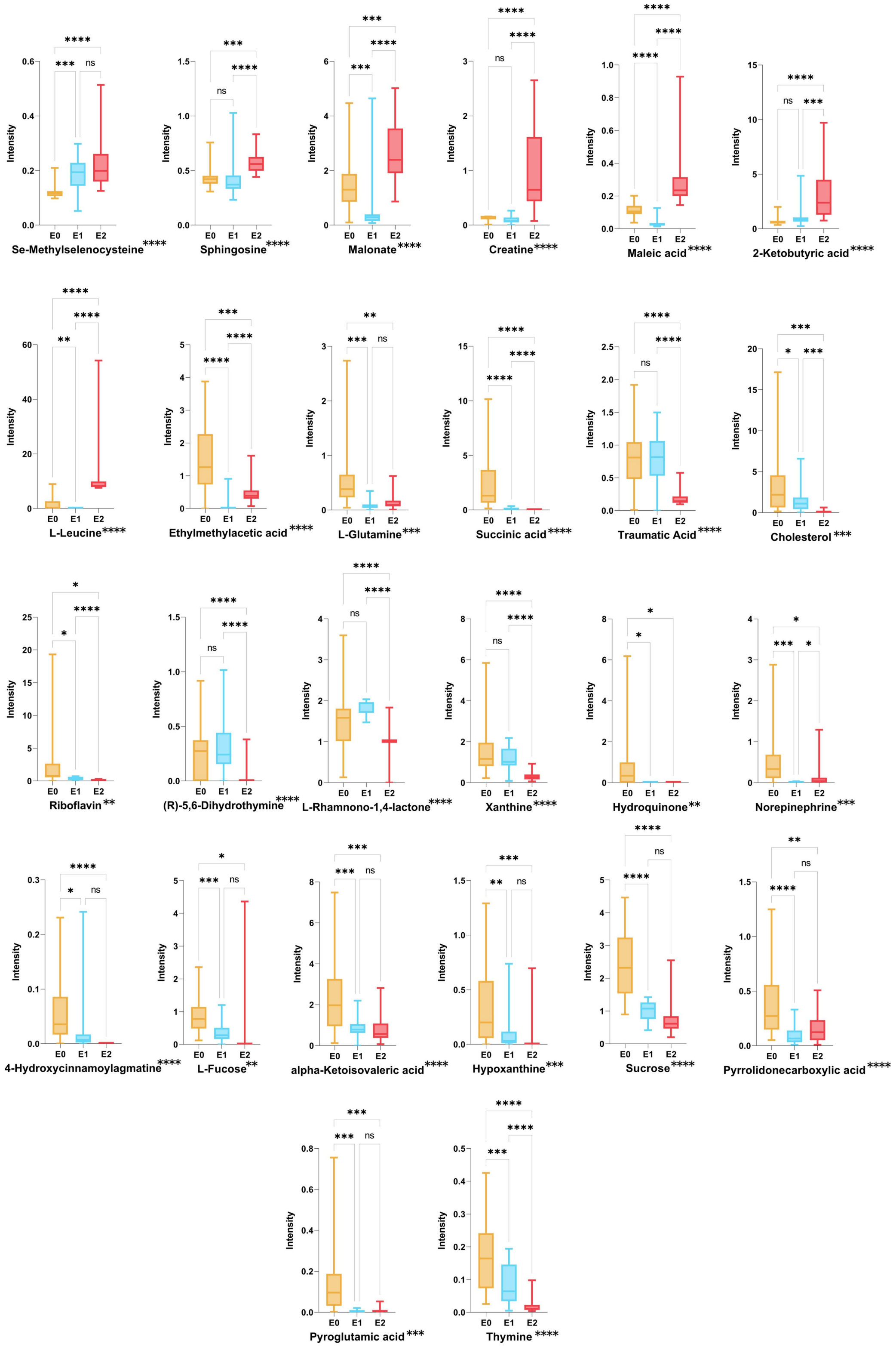

Figure S8 Changes of metabolites before and after closure of ileostomy. Repeated measurements of ANOVA were used to screen for DAMs, and Tukey test is used for multiple comparisons between groups. * means p value$<$0.05, ** means p value$<$0.01, *** means p value $<$0.001, **** means p value $<$0.0001, ns means no significant difference. **Abbreviation:** *ANOVA* analysis of variance, *DAM* differentially abundant metabolite.
